# Incessant Atrial Tachycardia From the Left Atrial Appendage Treated With Appendage Ligation

**DOI:** 10.1016/j.jaccas.2024.102375

**Published:** 2024-06-04

**Authors:** Yonathan F. Melman, Eric M. Lindley, Abishek Kashyap, Rami Alharethi, Peter Smit, Daniel Gutteridge, Joseph Palatinus, Annie Oesterling

**Affiliations:** a Department of Cardiology, McKay-Dee Hospital Ogden, Utah, USA; b Intermountain Medical Center, Murray, Utah, USA

**Keywords:** AtriClip, cardiogenic shock, ECMO, tachycardia mediated cardiomyopathy

## Abstract

A previously healthy man presented in shock due to incessant tachycardia. He ultimately required extracorporeal membrane oxygenation for support and clipping of his appendage for arrhythmia control. This case highlights the importance of early recognition of cardiogenic shock, aggressive hemodynamic support, and a multidisciplinary approach to managing these challenging arrhythmias.

## Graphical Abstract

**Figure:**
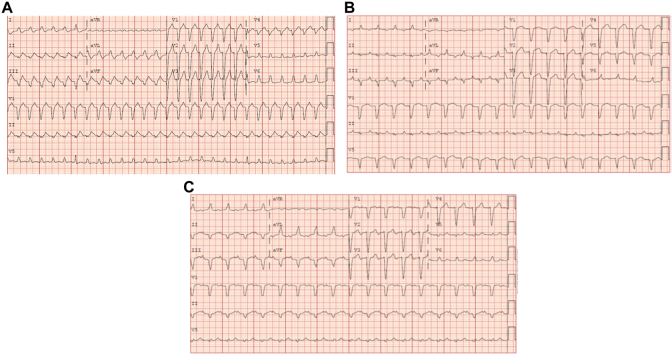

## History of Presentation

**Figure 1:**
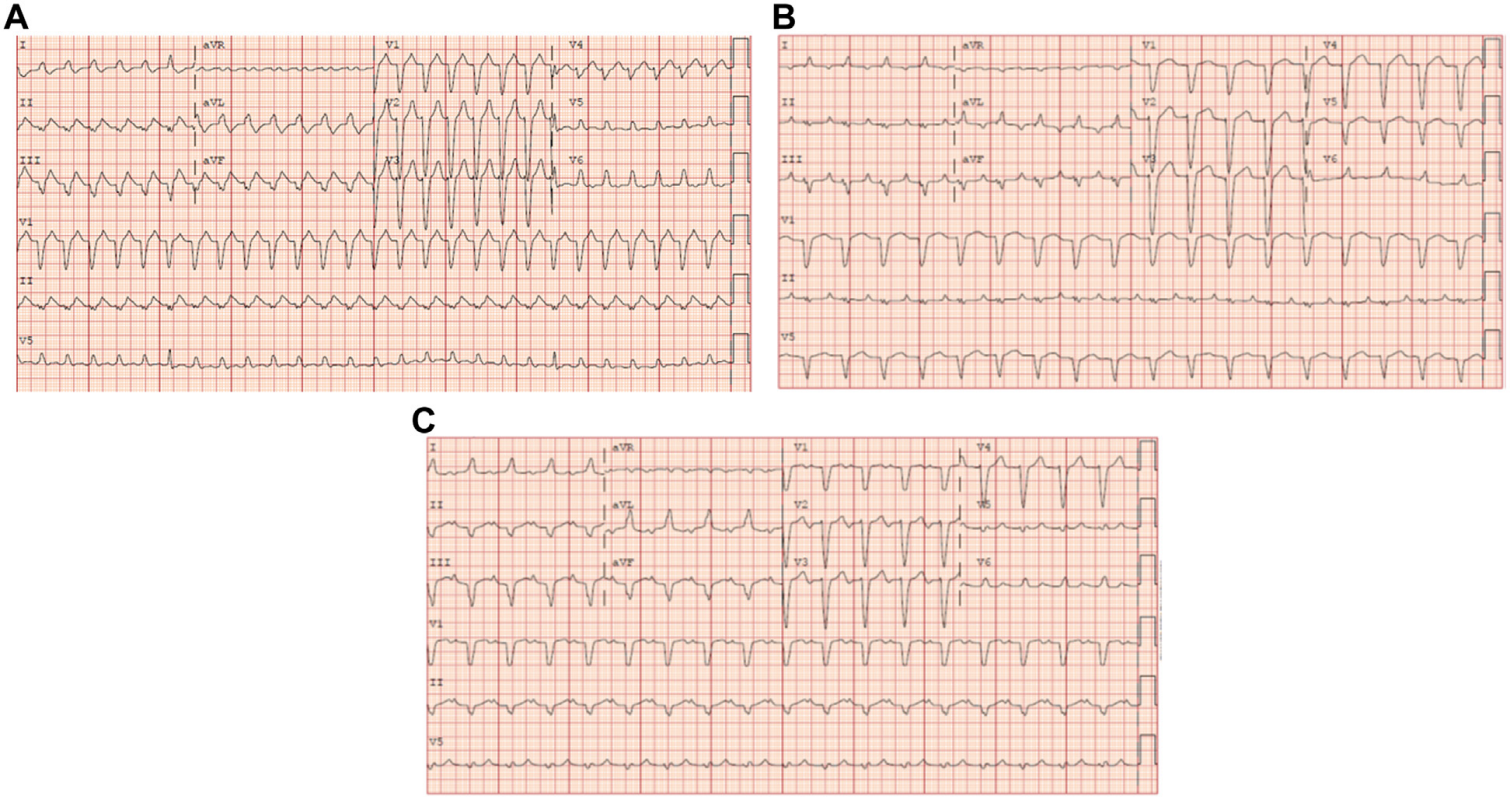
12-Lead Electrocardiogram During the Patient’s Admission (A) Twelve-lead of presenting rhythm. (B) Twelve-lead after ivabradine; note ectopic atrial focus with right inferior P-wave axis. (C) Normal sinus rhythm after appendage clipping.

A 46-year-old man presented to our institution in a wide complex tachycardia. He reported several months of worsening shortness of breath and feeling of heart racing. On arrival to an outside emergency department, his 12-lead electrocardiogram showed him to be in atrial tachycardia with left bundle branch block (Figure 1A). Cardioversion was attempted with prompt recurrence of arrhythmia shortly after cardioversion. He was given amiodarone bolus and infusion. Cardioversion was reattempted with recurrence, and he was transferred to our institution.

**Learning Objectives**

• To make a differential diagnosis for incessant tachycardia.
• To keep in mind the difficulty in managing incessant automatic atrial tachycardias that may require unconventional therapy with either ivabradine or surgical intervention.

## Past Medical History

He was an otherwise healthy, active man who had been able to exercise regularly until a few months prior to presentation. Of note, he did report a history of “tachycardia” diagnosed in his teens that required a cardioversion; however, no other information or records were available other than his own recollection of events.

## Differential Diagnosis

The differential diagnosis for young healthy patients with a wide complex tachycardia includes ventricular tachycardia vs supraventricular tachycardia with aberration, as this patient’s electrocardiogram suggested. Etiologies for tachycardia include bypass tract mediated atrioventricular reciprocation, atrioventricular nodal re-entry tachycardia, permanent junctional reciprocating tachycardia, and focal atrial tachycardia.

## Management

On arrival at our institution, he was noted to be persistently tachycardic. Vital signs were as follows: temperature 35 °C, heart rate 169 beats/min, blood pressure 115/85 mm Hg, respiratory rate 18, and oxygen saturation 99% on room air. He was hemodynamically stable and normotensive, with unremarkable laboratory results including troponin and lactate. N-terminal pro–B-type natriuretic peptide was 4,509 pg/mL. He was short of breath on lying flat, and reported in retrospect the insidious onset of symptoms over the prior few months. Echocardiogram was performed notable for a severely diminished ejection fraction at approximately 10%, with otherwise unremarkable valvular function and chamber size. The ejection fraction was globally decreased, with noted dyssynchrony in the setting of his wide left bundle branch block as a likely contributor to his myopathy.

**Figure 2:**
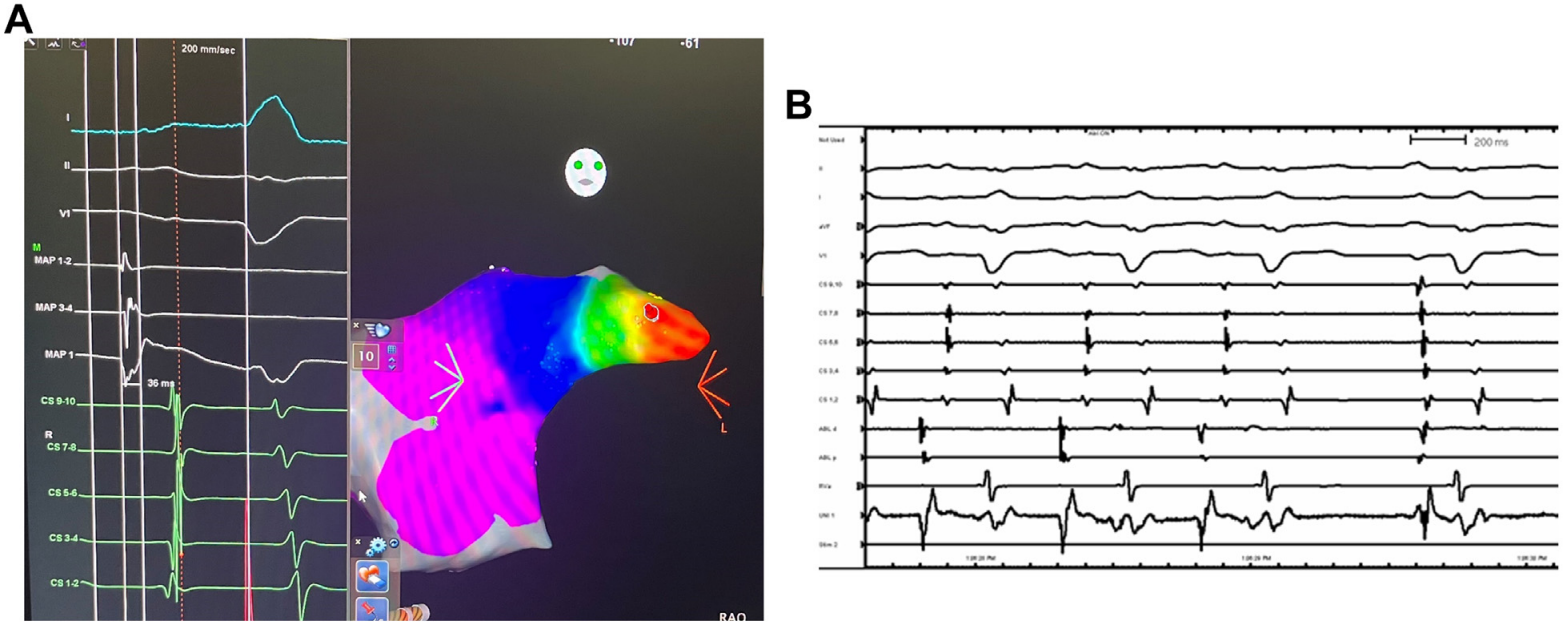
Mapping and Ablation of Tachycardia (A) Three-dimensional electroanatomical mapping using the Carto system localizes the tachycardia to the tip of the left atrial appendage. (B) Termination within 1 second of ablation at earliest site.

Given his drug refractory incessant atrial tachycardia and severely diminished ejection fraction, we made the decision to attempt catheter ablation. With cardiac anesthesia and the cardiac surgery team available for emergent hemodynamic support in case of decompensation, the patient was brought to the catheterization laboratory under light conscious sedation. The surface P-wave morphology was difficult to appreciate in the setting of tachycardia and wide left bundle branch block with secondary repolarization abnormality. However, activation in the coronary sinus was from distal to proximal. Ventricular overdrive pacing maneuvers confirmed the arrhythmia to be an atrial tachycardia. We then mapped a focal atrial tachycardia to the tip of the left atrial appendage. Earliest activation preceded the surface P-wave by approximately 35 milliseconds (Figure 2A). The unipolar signal was consistent with the catheter being near the site of origin. With the application of radiofrequency (RF) energy, there was termination to sinus rhythm within 1 second (Figure 2B). Lesions were applied at 10*g* to 20*g* of force, and 30 W of RF was used. Given the location deep within the appendage and the potentially catastrophic implications of a perforation, we were hesitant to apply force or RF power more aggressively, especially given the prompt termination. After termination, 3 additional lesions were placed for consolidation with similar settings. Approximately 4 hours postprocedure, he was noted to have recurrence of tachycardia. We attempted medical therapy with continued amiodarone loading and treatment of his heart failure. However, several hours later, he suffered severe decompensation with worsening shortness of breath, hypotension, a narrow pulse pressure to 80/70 mm Hg requiring Bilevel Positive Airway Pressure, and pressor support with minimal response.

As such, he was emergently placed on Cardiohelp peripheral cardiopulmonary support (Maquet Cardiopulmonary GmbH) and transferred to a quaternary care facility for further management. He required continuous infusions of dobutamine and norepinephrine in addition to mechanical support, and amiodarone intravenous infusion was continued with only brief periods of sinus rhythm. Angiography on hospital day 2 demonstrated normal coronaries and elevated left ventricular filling pressures; an Impella was placed for left ventricular venting. Cardioversion after several days of amiodarone loading was unsuccessful. On hospital day 3, a trial of ivabradine was attempted. Ivabradine slowed his tachycardia rate to 80 to 90 beats/min but did not terminate the arrhythmia (Figure 1B). At this rate, his ejection fraction and hemodynamics improved, with the ejection fraction coming up to the 40% range after 2 days of ivabradine therapy. On hospital day 5, he was decannulated from Cardiohelp; however, he remained in arrhythmia and his heart rate began to trend back up.

As such, on hospital day 7, he underwent thoracoscopic exclusion of the left atrial appendage using a 45-mm AtriClip. With application of the clip, there was immediate conversion to sinus rhythm (Figure 1C). With control of his heart rate, his clinical status stabilized and improved. His repeat ejection fraction on postoperative day 5 was 45%, and the Impella was removed. He was able to be discharged on hospital day 14 on a regimen of metoprolol, spironolactone, and valsartan with close follow-up arranged. At 1-month follow-up, he is completely asymptomatic. He has not had recurrence of tachycardia and monitors himself regularly with use of a Fitbit. He feels that his energy and stamina have returned to baseline.

## Discussion

We present a case of severe systolic dysfunction and cardiogenic shock due to an incessant atrial tachycardia that was refractory to cardioversion, ablation, amiodarone, and ivabradine and ultimately required left atrial appendage clipping for treatment. Focal atrial tachycardias can arise from anywhere within the 2 atria; however, locations within the pulmonary veins and appendages have a particularly high frequency of becoming incessant and more frequently cause tachycardia mediated cardiomyopathy than other foci.^1^ Both medical therapy and catheter ablation are established therapies for controlling focal tachycardias from the appendages.^2^

The use of ivabradine to control focal tachycardias has been reported in the literature,^3^, ^4^, ^5^, ^6^, ^7^, ^8^ with 1 larger case series documenting a significant percentage of treatment failure that requires catheter ablation for eventual control.^9^ Ivabradine works by inhibiting the “funny current” I_f_, which mediates phase 4 depolarization in pacemaker tissues.^10^ I_f_ is responsible for phase 4 depolarization in the sinoatrial and atrioventricular nodes, and has also been described in Purkinje cells in both atria and ventricles. It may explain the sensitivity of ectopic rhythms to the medication. In the largest published case series, a significant proportion of patients experienced transient or no response to the arrhythmia and required catheter ablation for definitive treatment of the arrhythmia.

In this case, ivabradine was ultimately unsuccessful in providing definitive arrhythmia control; nonetheless, the transient control of heart rate allowed for at least some myocardial recovery and hemodynamic stabilization. Our previous activation map had localized the focus of the tachycardia to be in the distal aspect of the left atrial appendage; we therefore decided to proceed with left atrial appendage clipping to exclude the tachycardia focus. Clipping of the appendage to treat a focal tachycardia has been reported only once before in the literature.^11^ The present case illustrates both the importance of prompt recognition and management of impending cardiogenic shock from tachycardia mediated cardiomyopathy and the use of pharmacologic, ablative, and surgical techniques in managing difficult-to-treat arrhythmias.

## Follow-up

Since discharge, the patient has not had recurrence of tachycardia and he monitors himself regularly at home. He reports complete resolution of all heart failure symptoms and is back to his baseline functional status. Most recent ejection fraction 5 days after tachycardia termination shows his ejection fraction in the low to normal range at 45%.

## Conclusions

In this young patient, an incessant focal atrial tachycardia caused a tachycardia mediated cardiomyopathy which led to cardiogenic shock. Aggressive early mechanical support and treatment with ivabradine allowed for sufficient stabilization and myocardial recovery and was able to go through an appendage clipping to control his arrhythmia.

## Funding Support and Author Disclosures

The authors have reported that they have no relationships relevant to the contents of this paper to disclose.
